# Multidimensional single-cell analysis identifies a role for CD2-CD58 interactions in clinical antitumor T cell responses

**DOI:** 10.1172/JCI159402

**Published:** 2022-09-01

**Authors:** Gabrielle Romain, Paolo Strati, Ali Rezvan, Mohsen Fathi, Irfan N. Bandey, Jay R T. Adolacion, Darren Heeke, Ivan Liadi, Mario L. Marques-Piubelli, Luisa M. Solis, Ankit Mahendra, Francisco Vega, Laurence J.N. Cooper, Harjeet Singh, Mike Mattie, Adrian Bot, Sattva S. Neelapu, Navin Varadarajan

**Affiliations:** 1 William A. Brookshire Department of Chemical and Biomolecular Engineering, University of Houston, Houston, Texas, USA.; 2 Department of Lymphoma and Myeloma and; 3 Department of Translational Molecular Pathology, The University of Texas MD Anderson Cancer Center, Houston, Texas, USA.; 4 CellChorus, Houston, Texas, USA.; 5 Kite, a Gilead Company, Santa Monica, California, USA.; 6 Department of Hematopathology and; 7 Division of Pediatrics, The University of Texas MD Anderson Cancer Center, Houston, Texas, USA.

**Keywords:** Immunology, Oncology, Cancer immunotherapy

## Abstract

The in vivo persistence of adoptively transferred T cells is predictive of antitumor response. Identifying functional properties of infused T cells that lead to in vivo persistence and tumor eradication has remained elusive. We profiled CD19-specific chimeric antigen receptor (CAR) T cells as the infusion products used to treat large B cell lymphomas using high-throughput single-cell technologies based on time-lapse imaging microscopy in nanowell grids (TIMING), which integrates killing, cytokine secretion, and transcriptional profiling. Our results show that the directional migration of CD19-specific CAR T cells is correlated with multifunctionality. We showed that CD2 on T cells is associated with directional migration and that the interaction between CD2 on T cells and CD58 on lymphoma cells accelerates killing and serial killing. Consistent with this, we observed that elevated CD58 expression on pretreatment tumor samples in patients with relapsed or refractory large B cell lymphomas treated with CD19-specific CAR T cell therapy was associated with complete clinical response and survival. These results highlight the importance of studying dynamic T cell–tumor cell interactions in identifying optimal antitumor responses.

## Graphical abstract

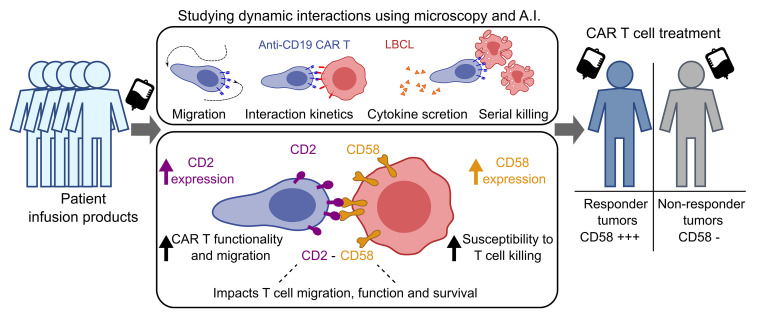

## Introduction

T cells stably endowed with a genetically encoded chimeric antigen receptor (CAR) targeting CD19 have shown clinical responses in refractory B-lineage leukemias and lymphomas. The potential for durable remissions has motivated the development of CARs targeting antigens other than CD19 for the treatment of hematological and nonhematological malignancies (1–3). While research on engineered T cells has focused on attributes that are common to the entire population of manufactured cells, such as tumor antigen discovery and immunoreceptor design (1, 4, 5), characterization of metrics of individual CAR T cells that define their functional potential, and thus clinical benefit, has not been adequately investigated.

Because of inter- and intratumor heterogeneity, technologies that aggregate T cell biology are unable to accurately capture the complexities of a T cell product with defined and desired characteristics. For example, it is widely accepted that less-differentiated cells have increased proliferative capacity and improved persistence, but individual cells vary in their persistence and functional potential (6–8). Correlative profiling of clinical infusion products (IPs) from responders and nonresponders to CAR T cell therapy has shown that products with less-differentiated, naive/memory-like T cells are enriched in complete responders, whereas signatures associated with T cell exhaustion (functional impairment) are enriched in nonresponders (9–13). While these results have advanced the field of CAR T cell biology, these studies do not directly profile the dynamic interactions between T cells and tumors and hence cannot identify the precise molecular interactions necessary for optimal antitumor function of CAR T cells.

## Results

**Figure 1:**
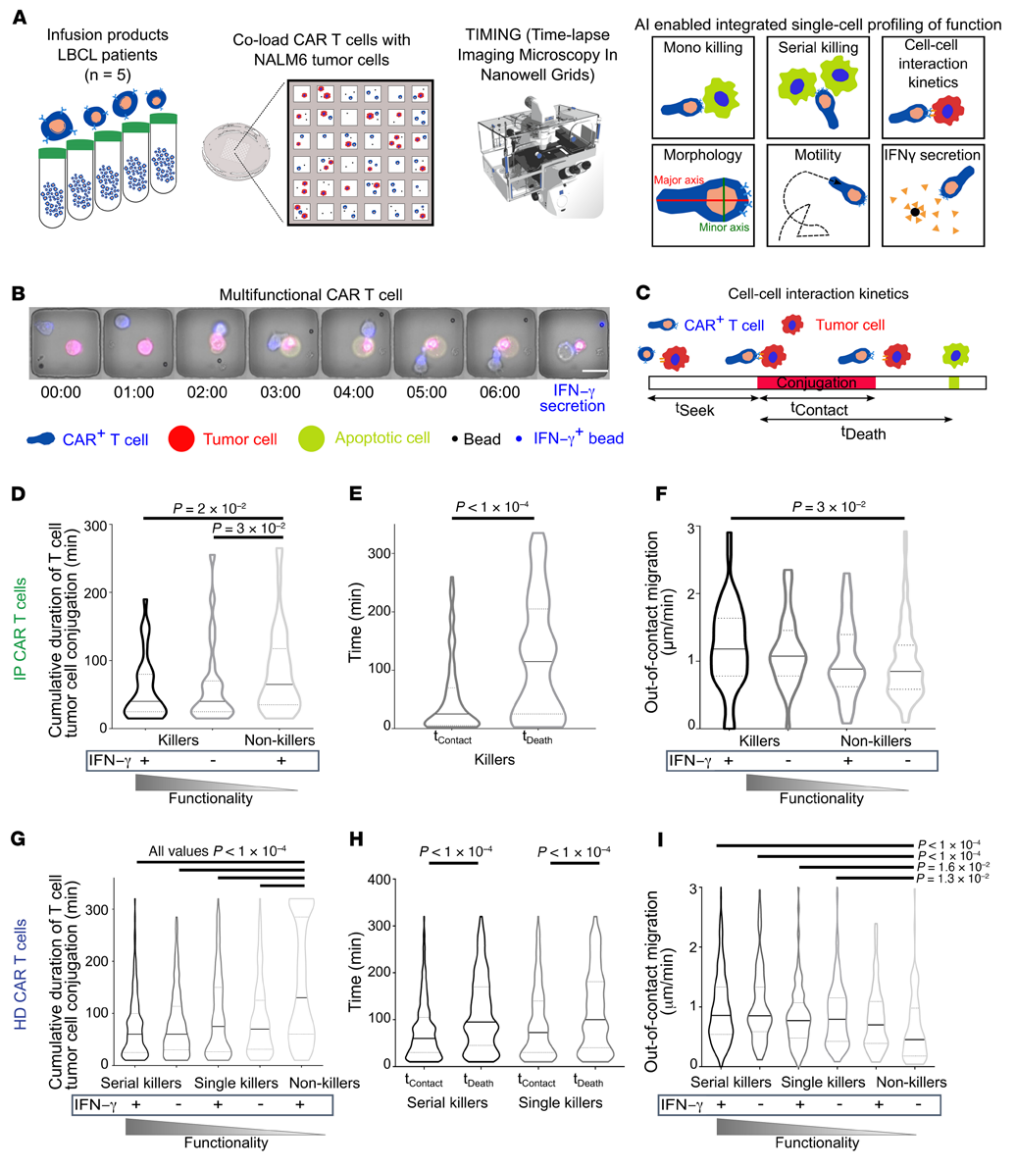
Dynamic single-cell profiling of the multifunctionality of CAR T cell IPs. (**A**) Schematic overview of the dynamic profiling and image analysis workflow of CAR T cell multifunctionality using TIMING. We evaluated the interaction between CAR T cells and NALM-6 cells as tumor cells on arrays of nanowells using TIMING. (**B**) A representative example of a multifunctional 19-28z T cell that participated in killing and secreted IFN-γ. TIMING is utilized to quantify T cell–intrinsic behavior such as directional migration and the kinetics of the interaction, leading to induction of apoptosis within tumor cells. After the TIMING assay, the IFN-γ molecules captured onto the beads during TIMING are revealed by using appropriate fluorescently labeled antibodies. Time is displayed as hours and minutes. Scale bar: 20 μm. (**C**) Schematic description of kinetic parameters measured in TIMING experiments. t_Seek_, time taken for the effector cell to conjugate to the tumor cell. (**D** and **G**) Cumulative contact duration between effector and tumor cells leading to different functional outcomes. Effector cells that only secrete IFN-γ (monofunctional) exhibited longer contact duration compared with cytolytic cells with or without IFN-γ secretion. Data are aggregated from profiling all 5 IPs. HD, healthy donor. (**E** and **H**) Comparative assessments of t_Contact_ and t_Death_ for all killer 19-28z T cells. (**F** and **I**) Out-of-contact migration of the different functional subsets of 19-28z T cells. All data in **D**–**F** correspond to E:T of 1:1 and are aggregated from profiling all 5 IPs (1589 T cells). All data in **G**–**I** correspond to an E:T of 1:2–5 (to evaluate serial killing) (1178 T cells). Each violin plot represents a minimum of 80 single cells. All *P* values for all multiple comparisons were computed using Kruskal-Wallis nonparametric tests and pairwise comparisons using a Mann-Whitney *U* test. Black bars represent the median, and the dotted lines denote quartiles.

We utilized time-lapse imaging microscopy in nanowell grids (TIMING) (14–16) to quantify the dynamic interactions between CAR T cells and tumor cells that lead to multifunctional T cell responses. We profiled IPs from 5 axicabtagene ciloleucel products (axi-cel; 19-28z CAR T cells [CD19-specific CAR construct with a CD8α spacer and CD28 and CD3-ζ endodomains]) comprising predominantly CD8^+^ T cells (54.5%–87.2 %) and memory cells (67.1%–82.3%) (Supplemental Figure 1; supplemental material available online with this article; https://doi.org/10.1172/JCI159402DS1). CAR T cells as effectors, NALM-6 tumor cells as targets, and prefunctionalized beads coated with IFN-γ capture antibody as cytokine sensors were loaded sequentially onto a nanowell grid array, and the kinetics of killing and end-point cytokine secretion from the same cells was monitored using TIMING (Figure 1, A and B, and Supplemental Video 1). We chose to track IFN-γ because of the known impact of CAR T cell–derived IFN-γ in shaping the susceptibility of tumors, enabling endogenous host immunity, and the potential for toxicity (17–19).

We classified the cells using trained convolution neural networks (CNNs) and performed cell segmentation and tracking using machine learning algorithms (Figure 1A). To define the kinetics of interaction between individual T cells and tumor cells that lead to subsequent killing, 2 interaction parameters, t_Contact_ (cumulative duration of conjugation between the first contact to target death) and t_Death_ (time between first contact and target apoptosis), were computed (Figure 1C and Supplemental Table 1 [glossary]). The frequency of multifunctional T cells exhibiting both killing and IFN-γ secretion was higher than that of cells that only secreted IFN-γ (monofunction) (Supplemental Figure 2). Furthermore, we observed that T cells that only secreted IFN-γ (monofunction) exhibited the longest conjugation duration of all functional (killing and/or IFN-γ secretion) T cells (Figure 1D). Second, for all killer T cells, t_Contact_ was significantly lower than t_Death_, demonstrating that T cell detachment preceded tumor cell apoptosis (Figure 1E). Collectively, these results profiling IPs showed that multifunctional T cells rapidly terminate synapses with tumor cells, whereas nonkiller T cells stay attached to tumor cells, leading to IFN-γ secretion without termination of the synapse.

We next investigated whether directional migration (direction of movement is maintained for at least 1 cell diameter, hereafter migration; Supplemental Video 2) can be associated with the efficient exploration of multiple tumor cells, leading to multifunctionality. T cells were classified into T cells migrating “out-of-contact” with tumor cells and “in-contact,” resulting in conjugation between T cells and tumor cells. Individual killer CAR T cells had higher out-of-contact migration in comparison with nonkiller T cells (Figure 1F). These observations were also consistent when measuring in-contact migration during conjugation with the tumor cell, wherein multifunctional cells had higher migration in comparison with nonfunctional cells (Supplemental Figure 3).

Unlike the small numbers of IP T cells available for profiling, 19-28z CAR T cells manufactured from healthy donors can be assayed in larger numbers. We next profiled 2 preclinical 19-28z CAR T cell products (>95% CAR^+^CD8^+^), specifically focusing on serial killing (20). We identified 1178 nanowells of interest populated with a single live T cell, 2 to 5 NALM-6 tumor cells, and 1 or more beads. Since every T cell within this subset was incubated with multiple tumor cells, 3 functional definitions were utilized: single killer cells, which killed only 1 tumor cell at an effector/target (E:T) ratio of 1:2–5; serial killer cells, which eliminated at least 2 tumor cells at an E:T ratio of 1:2–5; and monofunctional cells, which did not lyse targets, but only secreted IFN-γ. We confirmed that 2 observations were consistent with the clinical IPs. First, T cells that only secreted IFN-γ (monofunctional) exhibited the longest conjugation duration of all functional (killing and/or IFN-γ secretion) T cells. These differences were dominated by the presence of a subpopulation of T cells within the IFN-γ monofunctional cells that remained conjugated to the tumor cell for the entire period of observation (Figure 1G). Second, for both single killers and serial killers, t_Contact_ was significantly lower than t_Death_, demonstrating that T cell detachment preceded tumor-cell apoptosis (Figure 1H). Serial killer T cells had a lower duration of conjugation to tumor cells in comparison with monokiller T cells, independent of IFN-γ secretion (Supplemental Figure 4, A and B). Collectively, these results were similar to that in the IP T cells and showed that serial killer T cells rapidly terminate synapses with tumor cells, whereas nonkiller T cells stay attached to tumor cells, leading to sustained IFN-γ secretion.

We next confirmed that individual killer CAR T cells (both single and serial killers) had higher out-of-contact migration in comparison with nonkiller T cells (Figure 1I). Across the different functional subsets of T cells, as defined above, there was an association between decreasing out-of-contact migration and decreasing multifunctionality (Supplemental Figure 5). These observations were also consistent when measuring in-contact migration during conjugation with the tumor cell, wherein multifunctional cells and specifically serial killers had higher migration in comparison with the nonfunctional cells (Supplemental Figure 6, A and B). In aggregate, these results demonstrate that migratory T cells are multifunctional cells capable of killing and serial killing.

**Figure 2:**
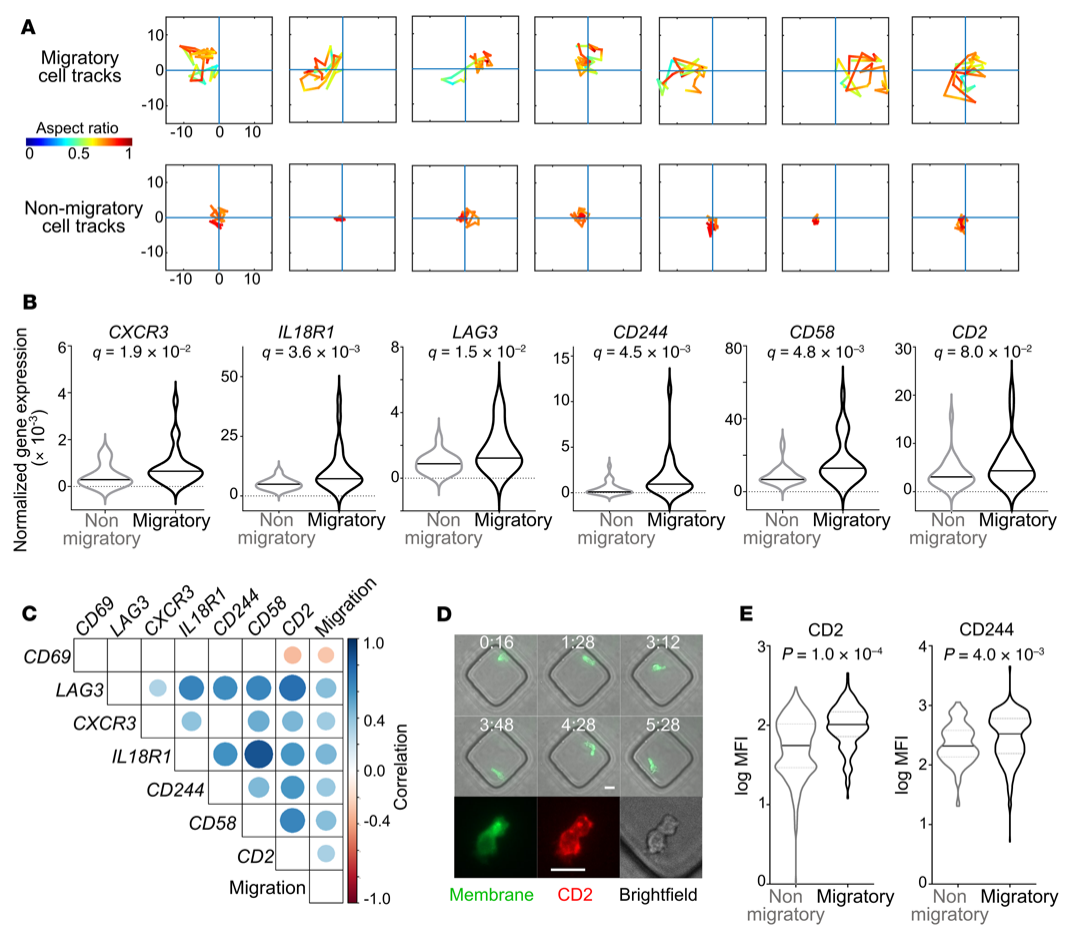
Biomarkers of directional T cell migration revealed by paired functional and single-cell transcriptional profiling. (**A**) Representative examples of high and low migration cell tracks during the 3-hour TIMING experiment. The *x* and *y* coordinates are shown in microns relative to the initial cell position set to the origin. Color map represents the aspect ratio of cell polarization with red denoting circular cells and increasing shades of green and blue denoting elongated cells. (**B**) Violin plots illustrating genes differentially expressed between the high- and low-migration 19-28z T cells. Genes that are differentially expressed at a FDR *q* < 0.1 are shown. For each group, data were derived from a minimum of 30 single cells. (**C**) Correlogram illustrating the pairwise correlation coefficients of the transcripts that are significantly linearly correlated with migration. The size of the circle reflects the strength of the correlation, and only the significant correlations (*P* < 0.05) are shown. (**D**) A representative example of a migratory T cell tracked using TIMING that was subsequently labeled immunofluorescently with the antibody directed against CD2. Scale bars: 10 μm. (**E**) The differential expression of proteins associated with increased migration of T cells, as determined by immunofluorescent microscopy. For each group, data were derived from a minimum of 200 single cells. *P* values were computed using Mann-Whitney *U* tests.

To gain an understanding of the mechanism linking migration and function, we integrated dynamic single-cell profiling with transcriptional profiling. We set up a TIMING assay with CAR T cells without the confounding influence of tumor cells so that we could identify biomarkers of intrinsic T cell migration. Single migratory or nonmigratory T cells were retrieved (Figure 2A and Supplemental Videos 2 and 3), and we performed direct quantitative PCR–based (qPCR-based) amplification, since it has higher sensitivity than single-cell RNA-Seq (scRNA-Seq) (20). Six genes were upregulated in migratory T cells: *CXCR3* and *IL18R1* (chemokine and cytokine receptors), *LAG3*, *CD244*, *CD58*, and *CD2* (inhibition/activation receptors) (Figure 2B). Notably, the expression of *CAR*, *FASLG*, and *GZMB* was no different between the 2 subsets of T cells (Supplemental Figure 7, A–C). *PRF1* showed a trend toward increased expression in the migratory T cells in comparison with the nonmigratory T cells, consistent with their increased cytotoxicity, but this was not significant (Supplemental Figure 7D). Building upon our recent report (20), these results suggest that the differences in killing capacity between CAR T cells cannot be explained by differences in the abundance of the transcripts of key cytotoxic molecules.

To investigate the relationship between the expression of these differentially expressed genes and cellular migration, we computed pairwise correlation coefficients (Figure 2C). All 7 genes continued to demonstrate a significant correlation to cellular migration, and CD2 transcripts were significantly correlated to all the other genes tested (Figure 2C). CD2 and CD244 are members of the CD2 family of proteins and interact with the partner proteins CD58 and CD48 either on other T cells (homotypic) or tumor cells (heterotypic). We quantified the basal migration of CAR T cells in the absence of tumor cells by TIMING and then quantified a posteriori the cell surface abundance of CD2 and CD244 by fluorescent immunostaining and microscopy (Figure 2D). Comparison of median fluorescence intensity showed that migratory cells had a significantly higher expression of both proteins in comparison with nonmigratory cells within the same CAR T cell populations (Figure 2E), and these results suggest that CD2 can function as a phenotypic biomarker for migratory T cells.

**Figure 3:**
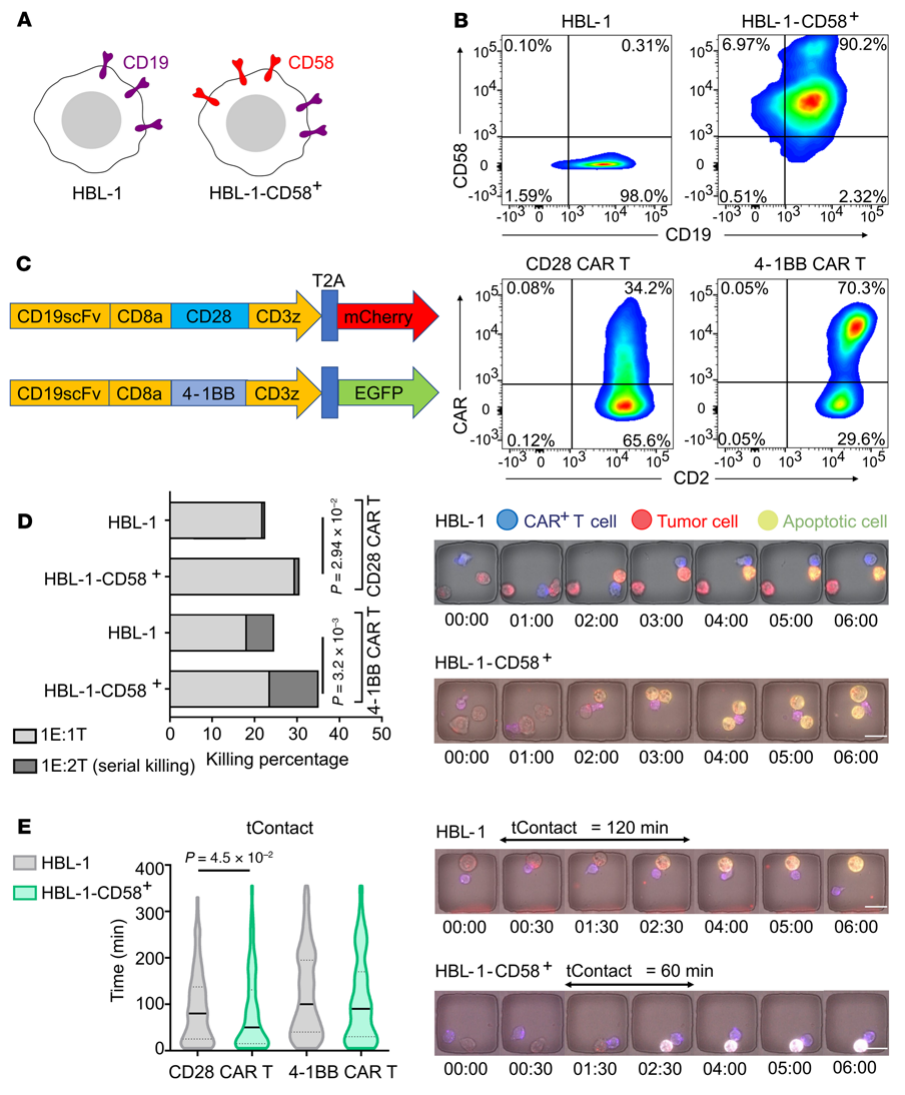
CD58 expression on DLBCL cells enables multifunctionality of CAR T cells. (**A**) The DLBCL cell line HBL-1 is deficient in CD58 expression due to homozygous deletion of the CD58 loci. The long form of CD58 was lentivirally transduced into HBL-1 cell lines. (**B**) Flow cytometric assays demonstrating the relative expression of CD19 and CD58 on HBL-1 and HBL-1–CD58^+^ cell lines. (**C**) Design of the CAR constructs and CD2 expression in 19-28z and 19-41BBz CAR T cells. (**D**) T cell–mediated killing of HBL-1 and HBL-1–CD58^+^ cells evaluated using TIMING (E:T 1:1–2). For each group, data were derived from a minimum of 150 single CAR T cells. *P* values were computed using 2-tailed Fisher’s exact test. Representative micrographs illustrating monokilling of HBL-1 cells and serial killing of HBL-1-CD58^+^ cells are shown. Time is displayed as hours and minutes. Scale bar: 20 μm. (**E**) Kinetic differences in the nature of interaction between CAR T cells and HBL-1 and HBL-1–CD58^+^ cells evaluated using TIMING (E:T 1:1). For each group, data were derived from a minimum of 150 individual CAR T cells. *P* values were computed using Mann-Whitney *U* test. Representative micrographs illustrating the duration of contact before killing between a 19-28z T cell and HBL-1 cell, and a 19-28z T cell and HBL-1–CD58^+^ cell. Time is displayed as hours and minutes. Scale bars: 20 μm. Black bars represent the median, and dotted lines denote quartiles.

To explore the significance of the interaction of these receptors during interaction with tumor cells, we blocked either CD58 (on NALM-6 targets) or CD2/CD244 (on CAR T cells). We did not block CD48, since leukemic cell lines, including NALM-6, do not express this protein(21). We observed that IFN-γ secretion was significantly reduced upon blocking either CD2 or CD58, but not CD244 (Supplemental Figure 8A). Next, we performed flow cytometry assays and confirmed that the number of degranulating CAR T cells was also reduced upon blocking CD58 on tumor cells (Supplemental Figure 8B). To understand the clinical implications of these results, we sought to investigate whether CD58 expression can affect killing mediated by CAR T cells in diffuse large B cell lymphoma (DLBCL). Accordingly, we expressed CD58 (long isoform) in the HBL-1 DLBCL cell line that lacks CD58 expression (Figure 3, A and B). We tested healthy donor–derived 19-28z and 19-41BBz CAR T cells (Figure 3C and Supplemental Figure 9) against parental HBL-1 (CD58^–^) and HBL-1–CD58^+^ cells using TIMING. Flow cytometric analyses confirmed that both sets of T cells had high expression of CD2 (Figure 3C). For both 19-28z and 19-41BBz CAR T cells, we observed higher killing against HBL-1–CD58^+^ cells in comparison with the parental HBL-1 cells (Figure 3D and Supplemental Videos 4 and 5). Kinetically, this was associated with a shorter duration of synapse formation prior to killing, and this ability to form efficient cytolytic synapses is consistent with the recently published mechanism of CD2 ligation in amplifying antigen-mediated primary signaling (Figure 3E) (22). In aggregate, these results show that the interaction between CD2 (T cells) and CD58 (tumor cells) promotes optimal antitumor cytolytic functionality.

We next sought to quantify the impact of CD2 and CD58 expression in patients with B cell malignancy (large B cell lymphoma [LBCL]) treated with standard-of-care axi-cel (a 19-28z CAR T cell product). CD2 and CD58 expression in the IP were quantified by scRNA-Seq (Gene Expression Omnibus [GEO] GSE151511) on CAR T cells (11). Consistent with our preclinical data, the majority of CD3^+^CAR^+^ T cells (either CD4 or CD8) for both patients achieving complete remission (CR) (59%–94%) and those with progressive disease (PD) (74%–98 %) were CD2^+^, and there was no significant difference between the groups (Supplemental Figure 10, A and B). Similarly, there was no significant difference between the CR and PD groups in terms of *CD58* expression (Supplemental Figure 11, A and B). In both CD8^+^CAR^+^ and CD4^+^CAR^+^ T cells, the expression of *CD2* was higher in the more differentiated T cell subsets (effector memory T cells [TEM cells] and effector memory cells reexpressing CD45RA [TEMRA cells]) compared with central memory T cells (TCM cells) and naive T cells (TN cells) (Supplemental Figure 12A). *CD58* expression, on the other hand, increased with T cell differentiation from TN through TEM cells; however, TEMRA cells expressed the lowest levels of CD58 (Supplemental Figure 12B).

**Figure 4:**
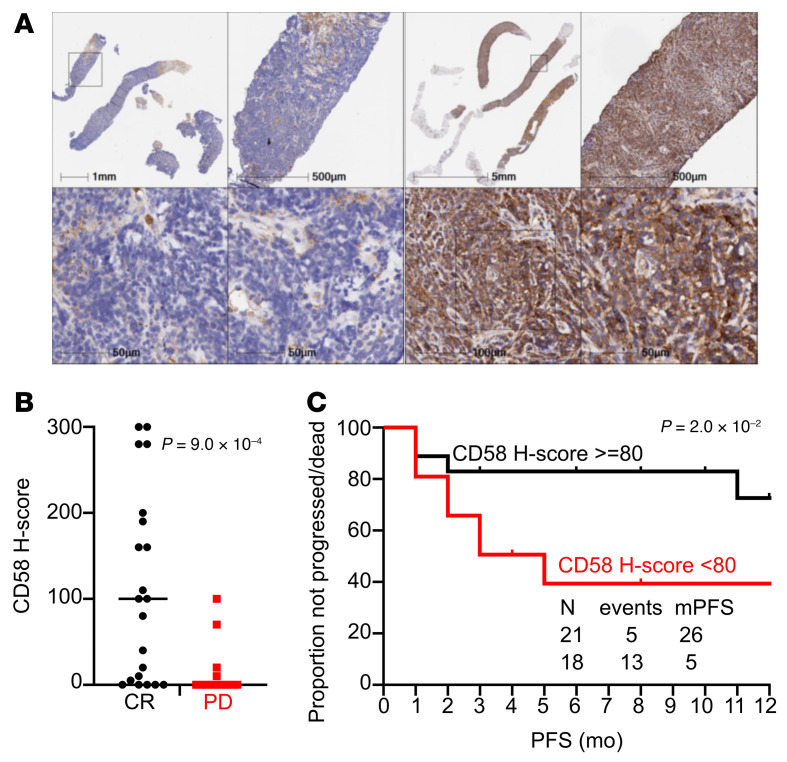
Prognostic role of pretreatment CD58 expression by IHC in patients with relapsed refractory LBCL treated with axi-cel. (**A**) CD58 expression by IHC in tissue samples collected before treatment with standard-of-care axi-cel. Left panels (negative case): IHC for CD58 shows diffuse negativity of the lymphoma cells with positive internal control (bars show the depth of magnification). Right panels (positive cases): IHC for CD58 shows diffuse cytoplasmic and membranous stain with strong intensity in all lymphoma cells (bars show the depth of magnification). (**B**) CD58 H score according to day 90 response to standard-of-care axi-cel. Bars represent the median. CR, *n* = 21; PD, *n* = 18. *P* value was computed using Mann-Whitney *U* test. (**C**) PFS after standard-of-care axi-cel according to pretreatment CD58 H score (cut-off = 80). N, number. PFS was calculated using Kaplan-Meier estimates and was compared between subgroups using the log-rank test.

Next, we assessed CD58 expression in tumor cells by chromogenic IHC in 39 patients with relapsed or refractory LBCL treated with standard-of-care axi-cel for whom pretreatment tissue biopsy was available. The baseline clinical characteristics of the patients are described in Supplemental Table 2. We observed a complete lack of expression in 12 (31%) cases (Figure 4A; H score = 0). In the remaining 27 (69%) patients, IHC for CD58 showed high intensity with diffuse membranous and cytoplasmatic staining, with a median H score of 110 (range, 5–300) (Figure 4A).

Twenty-one (54%) patients showed CR on PET-CT scan performed on day 90, whereas 18 (46%) were either primary refractory or relapsed/progressed by day 90 after initial response on day 30 (PD). A significantly higher median H score was observed in patients with ongoing CR at day 90 compared with those with PD (100 versus 7.5) (Figure 4B). After a median follow-up of 12 months (95% CI, 6–18 months), 18 (46%) patients had either progressed and/or died, and median progression-free survival (mPFS) was 14 months (95% CI, 8–20 months). A significantly shorter mPFS was observed when comparing patients with elevated pretreatment CD58 expression, defined as H score of 80 or more (determined by a receiver operating characteristic [ROC] curve corresponding to a specificity of 0.94), with those with lower expression (26 months versus 5 months, *P* = 2 × 10^–2^) (Figure 4C). Collectively, these results demonstrate the importance of pretreatment CD58 expression as a predictive biomarker of response to CAR T cells.

## Discussion

Our data illustrate the impact of directly studying dynamic interactions between T cells and tumor cells in enabling clinically relevant discoveries. Our study of the multifunctionality of CAR T cells showed that at the single-cell level, nonkiller, IFN-γ–secreting cells have extended periods of conjugation with tumor cells. The existence of a subpopulation of CAR T cells that remain continuously conjugated to tumor cells due to lack of killing and leading to IFN-γ secretion suggests a hypothesis for CAR engineering. Since the functional affinity of the CAR is the primary determinant of the duration of conjugation between the T cell and the tumor cell, engineering CAR designs with altered binding kinetics should identify CARs with preserved multifunctionality but decreased IFN-γ secretion. This is clinically relevant, since chronic IFN-γ secretion is known to be associated with neurotoxicity after CAR T cell infusions (23). Indeed, altered CAR designs with lower affinity for CD19 or humanized CARs with a CD8α hinge have shown robust clinical responses with only minimal serum IFN-γ and associated neurotoxicity (24, 25).

The integrated transcriptional, phenotypic, and functional single-cell data demonstrated a correlation between CD2 (LFA-2) and CD58 (LFA-3) and directional migration. Although the positive significance of CD2 is highlighted from pan-cancer studies, it was inferred to merely reflect the presence of infiltrating lymphocytes (26, 27). Our combined functional, transcriptional, and phenotypic data advance the role of CD2-CD58 interactions at the single-cell level and are complementary to independent cell-cell interaction mechanistic studies probing the genes/proteins essential for cancer immunotherapy using CRISPR-Cas9 screens that mimic loss-of-function mutations involved in resistance to these therapies or in CRISPR-Cas9 screens that identify molecules essential for cytokine secretion (28, 29).

The CD2-CD58 interaction can influence each of the steps that determine the efficacy of infused T cells: migration, function, and survival. T cells after infusion need to traffic to the site of the tumor. CD58 is one of the T cell costimulatory molecules expressed by endothelial cells (ECs), and the CD2-CD58 interaction between T cells and ECs can facilitate recruitment of the circulating T cells to the site of the tumor (30). Once the T cells traffic to the tumor microenvironment, they are likely to encounter tumor cells expressing inhibitory molecules such as programmed death-ligand 1 (PD-L1). Within this context, in vitro studies support the concept that while programmed cell death protein 1 (PD-1) engagement on T cells can cancel costimulation by CD28, costimulation by CD2 is less sensitive to PD-1 inhibition (31). Not surprisingly, CD8^+^ tumor-infiltrating lymphocytes (TILs) in human cancers are characterized by a quantitatively lower expression of CD2, and CD2 mRNA levels in these cells are negatively correlated with exhaustion (22). Independently, it has been shown that CD2 can activate T cells without leading to exhaustion, as indicated by the lack of induction of inhibitory receptors, including PD-1 (32). T cells are also sensitive to activation-induced cell death (AICD), principally mediated by the ligation of Fas/Fas-L. The binding of CD58 to CD2 on activated T cells protects these T cells from AICD by blocking Fas/Fas-L upregulation, leading to survival and sustained antitumor efficacy (33, 34).

From a clinical perspective, the loss of CD58 has been associated with relapse and immune escape in hematological cancers and thus has broader impact beyond just CAR T cell therapy, as we have outlined here (35–37). Immune checkpoint blockade (ICB) therapies have had unprecedented clinical success across diverse cancer types (38). Yet the majority of patients are resistant to ICB therapy, and understanding mechanisms of resistance is an ongoing clinical need. scRNA-Seq of melanoma patient tumor ecosystems identified a baseline gene signature that included downregulation of CD58 as associated with T cell exclusion (cold tumors) and intrinsic resistance to ICB therapy (39). Independently, expression levels of *CD58* mRNA were significantly lower in melanoma patients that failed ICB therapy compared with treatment-naive patients (40). Mechanistic studies with expanded TILs have demonstrated that CD58-knockout melanoma cells are less susceptible to killing by TILs and NK cells, which is similar to our observations with CAR T cells (40). Bispecific T cell engagers (BiTE) redirect cytolytic function of T cells toward cancer cells and are being tested clinically against multiple solid tumors (41). Preclinical data from animal models support that loss of CD58 led to significantly decreased BiTE-mediated cytotoxicity and consequently antitumor efficacy (42). Both in the context of ICB and BiTE, it is clear that costimulation through CD28 and CD2 can function through nonoverlapping and synergistic mechanisms (42–44). The loss of CD58 expression within the tumors enables escape and resistance to immune cell attack, leading to treatment failures.

A recent conference abstract reporting on patients with DLBCL treated with axi-cel showed that tumor CD58 carried defects in 24% of the evaluated subjects (45). These defects were associated with a drastically reduced durability of response to axi-cel. Coexpression of CD2 in trans but not cis, relative to the CAR, restored the functional capability of CAR T cells when CD58 was disabled. In addition to corroborating our evidence presented here, these results suggest that the CD2-CD58 findings can be investigated along several paths. From a diagnostic perspective, patients can be stratified based on CD58 expression and monitored to determine whether escape from adoptive cell therapy is accompanied by the expansion of CD58-negative tumor cells (46, 47). From a CAR therapeutic perspective, incorporation of CD2 expression with functional signaling would be predicted to facilitate antitumor responses independently of CD58 on DLBCL (48). Therapeutic strategies to restore CD58 expression in tumors with downregulated CD58 expression (but not genetic loss) provide a path for directly improving clinical outcomes for ICB-resistant tumors, BiTE treatment, and treatment with expanded TILs for cell therapies. Drugs such as the immunomodulatory drug lenalidomide or inhibitors of the epigenetic modulator EZH2 are promising leads, since they have been shown to restore CD58 expression in cancer cells (49, 50).

## Methods

### Cell lines.

Human pre–B cell leukemic line NALM-6 cells (ATCC) were cultured in T cell medium (RPMI plus 10% FBS) and used as CD19^+^ target cells. The HBL-1 DLBCL cell line expressing CD58 was prepared as described previously (35). Parental HBL-1 and HBL-1–CD58^+^ were cultured in IMDM plus 10% FBS.

### Manufacture of CAR T cells.

We generated CAR T cells as previously described (20). Briefly, we activated PBMCs using OKT3 and anti-CD28 antibodies for 48 hours. Next, we transduced the activated T cells with retroviral particles of either CD19R-CD28 or CD19R-4-1BB CAR in RetroNectin-coated 24-well plates (Takara Bio). The CAR T cells were supplemented with cytokines IL-7 and IL-15. On day 10, we validated the CAR expression with a homemade anti-CAR antibody (51) using flow cytometry. All in vitro studies were performed on 10-day-old CAR T cells.

### Bead preparation: coating beads with the primary capture antibody.

Goat anti-mouse IgG-Fc beads (1 μL, ~2.3 × 10^5^ beads, Thermo Fisher Scientific) in solution were washed with 10 μL of PBS and resuspended in 19.6 μL PBS (~0.05% solids). Mouse anti-human IFN-γ (Mabtech, clone 1-D1K) was then added to beads at a final concentration of 10 to 40 μg/mL and incubated for 30 minutes at room temperature, followed by washing and resuspension in 100 μL PBS.

### Nanowell array fabrication and cell preparation.

We fabricated nanowell arrays for interrogation of effector functions at a single-cell level as described previously (52). Approximately 1 million effector cells and target cells were both spun down at 400*g* for 5 minutes, followed by labeling with 1 μM PKH67 and PKH26 fluorescent dyes (Sigma-Aldrich), respectively, according to the manufacturer’s protocol. Excess unbound dyes were then washed away, and cells were resuspended at approximately 2 million cells/mL concentration in complete cell-culture media (RPMI plus 10% FBS).

### TIMING assays for the multiplex study of effector cytolytic phenotypes and IFN-γ secretion.

We loaded capture antibody-coated beads and labeled effector and target cells sequentially onto nanowell arrays. Next, detection solution containing annexin V–Alexa Fluor 647 (annexin V–AF647) (Life Technologies) (for detection of target apoptosis) was prepared by adding 50 μL of stock solution to 2.5 mL of complete cell-culture media without phenol red. We imaged the nanowell arrays for 6 hours at an interval of 5 minutes using a LEICA/ZEISS fluorescent microscope utilizing a ×20 0.80 NA objective and a scientific CMOS camera (Orca Flash 4.0, version 2). At the end of the time-lapse acquisition, biotinylated mouse anti-human IFN-γ antibody (Mabtech, clone 7-B6-1) was added to 2.5 mL cell media at 1:1000 dilution. We subsequently incubated the array for 30 minutes, followed by washing and incubation with 5–10 μg/mL streptavidin conjugated to either R-phycoerythrin (PE) or AF647 (BioLegend). We imaged the entire chip to determine the intensity of the PE/AF647 signal on the microbeads, and the 2 data sets were matched using custom informatics algorithms (51).

### Image processing, cell segmentation, cell tracking, data analytics, and functional annotation.

Image analysis and cell segmentation/tracking were performed as described previously (16). The pipeline of image processing and cell segmentation ends with statistical data analysis based on the tabular spatiotemporal measurement data generated by the automated segmentation and cell-tracking algorithms. Within all nanowells that contained a single effector and 1 to 5 tumor cells, we identified nanowells in which the effector formed a conjugate (stable contact between effector cell and target cell lasting > 5 minutes). These nanowells containing effectors and targets with conjugation set the baseline for all our observations. We then partitioned these nanowells based on the functionalities of the cells, as follows: (a) multifunctional cells: T cells that participated in serial killing (regardless of IFN-γ secretion) or T cells that killed exactly 1 target cell and secreted IFN-γ; (b) monofunctional cells: T cells that performed only killing but no IFN-γ secretion or T cells that only secreted IFN-γ but did not kill; (c) nonkiller T cells: T cells that did not kill any tumor cells despite evidence of conjugation; and (d) nonfunctional cells: T cells that did not kill any tumor cells and did not secrete IFN-γ despite evidence of conjugation.

A size-exclusion filter based on maximum pixel areas was used to effectively differentiate cells from beads (beads were much smaller than cells). Where specified, cell tracks were represented using MATLAB, version 9.1 (Mathworks Inc.).

### Gene expression profiling.

PKH67-stained CD8^+^ T cells were loaded on a nanowell array, immersed with annexin V–AF647 containing phenol red–free complete cell-culture medium, and imaged for 3 hours using TIMING exactly as described above. After carefully washing the cells on the chip 3 times with cold PBS (4°C), cells were kept at 4^°^C until retrieval. Time-lapse sequences were manually analyzed to identify live cells with high and low migration. The cells were individually collected using an automated micromanipulating system (CellCelector, ALS) and deposited in nuclease-free microtubes containing 5 μL of 2× CellsDirect Buffer and RNase Inhibitor (Invitrogen). Single-cell reverse-transcription qPCR (RT-qPCR) was then performed using the protocol ADP41 developed by Fluidigm. Ninety-two cells (48 migratory and 44 nonmigratory) were assayed along with bulk samples of 10 and 100 cells and with no-cell and no-RT controls. The panel of 95 genes included genes relevant to T cell activation, signaling, and gene regulation and was designed and manufactured by Fluidigm D3 AssayDesign (20). Data analysis and processing were performed exactly as described recently (20).

### Flow cytometry–based phenotyping, cytokine secretion, and cytotoxicity assay.

For phenotyping, CAR T cells were stained using a panel of human-specific antibodies as follows: CD107a (clone H4A3), CD2 (clone RPA-2.10), CD58 (clone 1C3), CD244 (clone 2-69), CD62L (clone DREG-56), CD45RA (clone HI100), CD45RO (clone UCHL1), CD95 (clone DX2), CD3 (clone SK7), CD27 (clone L128, MT271), CD28 (clone L293), CD25 (clone M-A251), CD127 (clone HIL-7R-M21), KLRG1 (clone 2F1/KLRG1), and CD57 (clone NK-1) (all from BioLegend). CD4 (catalog OKT4), CD8 (catalog RPA-T8), CD69 (catalog FN50), and CCR7 (catalog G043H7) were from BioLegend. The anti-CAR scFv was made in house (53). To confirm CD58 expression on HBL-1 cells, they were stained with human-specific antibodies CD58 (clone 1C3) and CD19 (clone HIB19) from BD Biosciences. To assay cytotoxicity of the cells at the population level, NALM-6 target cells were stained with PKH26 and cocultured with T cells in triplicate conditions at different T cell–to–target cell ratios, with 100,000 T cells per well. Fluorescently labeled anti-CD107a antibodies together with GolgiStop (BD Biosciences) at 0.7 μL/mL was added to the coculture to stain for degranulating cells. After the assay, cells were washed and stained with Zombie Aqua (BioLegend) antibodies against CD4, CD8, CD3, and CD69, then analyzed by flow cytometry. To measure cytokine release in the supernatants of the cocultures, IFN-γ and TNF-α were quantified using the MultiCyt QBeads Human PlexScreen (Intellicyt) following the manufacturer’s protocol. For the blocking experiments, CAR T cells were incubated for 24 hours in flat-bottom 96-well plates that were precoated overnight with 10 μg/mL of purified anti-CD244 (clone C1.7, BioLegend), CD2 (clone LT2, Miltenyi), or anti-CD58 (clone 1C3, BD Biosciences) and rinsed once with complete culture medium before performance of the functional assay.

### TIMING and confocal microscopy.

CAR T cells were seeded onto nanowell arrays and the migration of the cells monitored using TIMING. At the end of 2 hours, the cells on the chip were washed carefully 3 times with cold PBS–5% FBS (4°C). Fluorescently conjugated monoclonal antibodies against CD2 (clone RPA-2.10, BioLegend), CD244 (clone 2-69, BioLegend), or CD58 (clone 1C3, BioLegend) were added to the chip, incubated for 30 minutes at 4^o^C, washed, and imaged on a Nikon Ellipse TE confocal microscope fitted with a ×60 0.95NA objective.

### Patient selection and response assessment.

All patients with relapsed or refractory LBCL treated with standard-of-care axi-cel at MD Anderson Cancer Center between 01/2018 and 04/2020 were included. The data cut-off for follow-up was on 10/20/2020. Response status was determined by the Lugano 2014 classification (54). Pretreatment tissue biopsies were collected for 21 patients who achieved CR after CAR T cell therapy and for 21 patients who were primary refractory to CAR T cell therapy (3 showed crush artifacts and could not be analyzed).

### CD58 validation, staining, and analysis.

Tissue sections of 4 μm thickness were prepared from formalin-fixed, paraffin-embedded (FFPE) lymph node and extranodal tissues and stained using Leica Bond RX automated stainer (Leica Biosystems). The antigen retrieval was performed with Bond ER Solution #1 (Leica Biosystems) equivalent to citrate buffer, pH 6.0 for 20 minutes at 100°C. Primary antibody against CD58 (CD58/LFA-3 goat polyclonal, R&D Systems) was used with a concentration of 1:100 (2 μg/ml) and incubated for 15 minutes at room temperature. The antibody was detected using the Bond Polymer Refine Detection Kit (Leica Biosystems) with diaminobenzidine (DAB) as the chromogen. All the slides were counterstained with hematoxylin, dehydrated, and coverslipped. Tonsils were used as external positive controls, and reactive immune cells were used as an internal control. CD58 expression was restricted mainly to neoplastic cells. Each case was analyzed using standard microscopy by 3 pathologists (blinded to response data), and the percentage (10% increments) and intensity (mild, moderate, and intense) of tumor cells staining were evaluated. The data were recorded using the H score system: (H score = [(% mild intensity × 1) + (% moderate intensity × 2) + (% intense intensity × 3)]).

The difference in a continuous variable between patient groups was evaluated by the Mann-Whitney *U* test. PFS was defined as the time from the start of axi-cel infusion to the progression of disease, death, or last follow-up (whichever occurred first). PFS was calculated using Kaplan-Meier estimates and was compared between subgroups using the log-rank test. ROC analysis was used to identify the optimal CD58 H score for survival analysis (H score = 80, specificity of 0.94). *P* < 0.05 (2 tailed) was considered statistically significant. Statistical analyses were completed using SPSS 24 (IBM) and Prism, version 8 (GraphPad).

### CD2 and CD58 expression in patients with B cell malignancy (LBCL).

Analysis was performed on scRNA-Seq (GEO GSE151511) of CAR T cells (11). Gene expression tables were uploaded into R (version 4.0) for processing using Seurat Package (version 4.0) (55). Following the standard workflow of the Seurat, we ended up with 30,000 CD3^+^CAR^+^ T cells from 22 patients (9 CR and 13 PD).

### Statistics.

Statistical analysis and plotting the data were performed in GraphPad Prism, version 8, and R, version 4.0. Schematics were made in Inkscape, version 1.1. Student’s *t* tests were 2-tailed, ANOVA tests were 1-way, and *P* < 0.05 was considered significant.

### Study approval.

All work outlined in this report was performed according to protocols approved by the Institutional Review Boards at the University of Houston and the University of Texas MD Anderson Cancer Center and conducted in accordance with the principles of the Declaration of Helsinki.

## Author contributions

GR, SSN, LJNC, HS, and NV designed the study. NV, GR, SSN, PS, AR, MF, and LJNC prepared the manuscript. GR, IL, JTA, INB, HS, PS, AM, MLMP, LMS, AR, and MF performed experiments. JTA, GR, NV, IL, PS, MLMP, LMS, AR, and MF analyzed data. HS, LJNC, SSN, FV, AB, MM, and DH provided patient samples. All authors edited and approved the manuscript.

## Supplementary Material

Supplemental data

Supplemental video 1

Supplemental video 2

Supplemental video 3

Supplemental video 4

Supplemental video 5

## Version 1 07/26/2022

In-Press Preview

## Version 2 09/01/2022

Electronic publication
