# Supplementary material for: Multidimensional single-cell analysis identifies a role for CD2-CD58 interactions in clinical antitumor T cell responses
Source: J Clin Invest. 2022 Sep 1;132(17):e159402. doi: 10.1172/JCI159402 (PMC9433104; doi:10.1172/JCI159402)
Supplement: Supplemental data [file jci-132-159402-s021.pdf]

Supplementary Information.

Multidimensional single-cell analysis identifies a role for CD2-CD58 interactions in clinical antitumor T cell responses

Gabrielle Romain, Paolo Strati, Ali Rezvan, Mohsen Fathi, Irfan N Bandey, Jay R T Adolacion, Darren Heeke, Ivan Liadi, Mario L Marques-Piubelli, Luisa M. Solis, Ankit Mahendra, Francisco Vega, Laurence J.N. Cooper, Harjeet Singh, Mike Mattie, Adrian Bot, Sattva Neelapu, and Navin Varadarajan

Table ST1. Glossary of terms used in the paper

CAR	Chimeric Antigen Receptor
Directional migration	Motion wherein the direction of movement is maintained for at least one cell diameter
19-28z	CD19-specific CAR construct with a CD8α spacer and CD28 and CD3-ζ endodomains
LBCL	Large B-cell lymphoma
DLBCL	Diffuse large B-cell lymphoma
Axi-cel	Axicabtagene ciloleucel
E	Effector CAR T-cell
T	Target cell
TIMING	Timelapse Imaging Microscopy In Nanowell Grids
Monofunction	The ability of a single T-cell to exhibit exactly one function (killing only or IFN-γ secretion only)
Multifunctional	The ability of a single T-cell to kill multiple tumor cells (with or without IFN-γ secretion) or kill exactly one tumor cell and secrete IFN-γ
Single-killing	The ability of a single T-cell to kill exactly one target cells
Multi-killing	The ability of a single T-cell to kill two or more target cells
Single-killer	T cells that kill exactly one tumor cell at an E:T ratio of 1:2-5
Serial killer	T cells that kill at least two tumor cells at an E:T ratio of 1:2-5
Non-killer	T cells that conjugate to one or more tumor cells but failed to kill any of them
Killing efficiency	Description of the kinetics of killing mediated by individual T cells (Please see t_{Death} below)
Conjugation	Stable contact between effector cell and target cell lasting > 5 minutes
t_{Death}	Time elapsed between first conjugation and tumor cell apoptosis (Annexin V staining positive)
$t_{Contact}$	Cumulative duration of conjugation between synapse formation and t_{Death}
AR	The aspect ratio of polarization represented as the ratio of the minor and major axes of the cell fitted to an ellipse
d_{Well}	The net displacement of the T-cell centroid, within the nanowell, averaged over 5-minute intervals

Table ST2. Patient baseline clinical characteristics collected before initiation of lympho-depleting chemotherapy (day -5).

Patients (N=39)	Median [Range]<br>Number (%)
DLBCL/HGBCL, N (%)	31 (79)
Age (years)	58 [18-84]
Male, N (%)	28 (72)
ECOG performance status > 0, N (%)	31 (79)
Ann Arbor Stage III-IV, N (%)	33 (85)
IPI score 3-4, N (%)	19 (49)
C-reactive protein (mg/L)	29 [0.4-371]
Ferritin (mg/L)	855 [36-9949]
Lactate dehydrogenase > ULN, N (%)	26 (67)
Previous therapies (number)	4 [2-7]
Refractory disease, N (%)	29 (74)
Previous autologous SCT, N (%)	9 (23)

DLBCL, diffuse large B-cell lymphoma; HGBCL, high-grade B-cell lymphoma; comparator: transformed follicular lymphoma and primary mediastinal B-cell lymphoma; ECOG, Eastern Cooperative Oncology Group; IPI, internal prognostic index; SCT, stem cell transplant; N, number; ULN, upper limit of normal

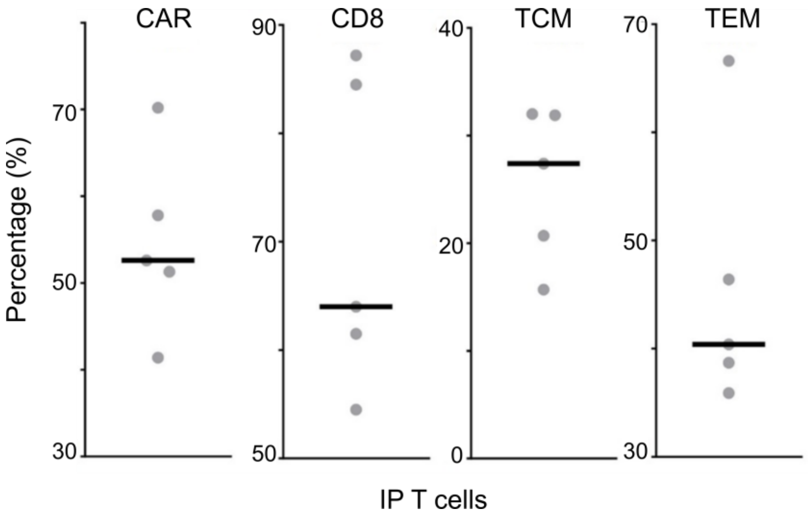

Supplementary Figure S1. **Phenotypic characteristics of the five IP CAR T cells profiled using TIMING, determined by flow-cytometry**. TCM and TEM are defined by flow cytometry by immunofluorescent staining of CD3+CD8+CAR+ cells using CD45RA and CD62L.

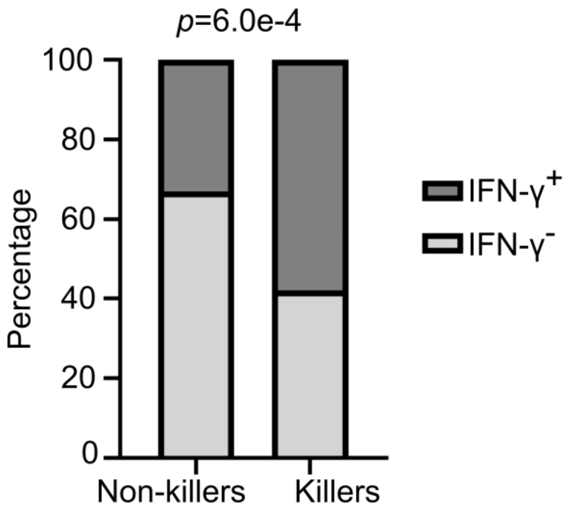

Supplementary Figure S2. Killer CAR T-cells secrete IFN-γ at higher frequencies compared to non-killer cells.

Comparisons of the IFN-γ secretion between killer and non-killer CAR-T cells (infusion products). Data was derived from 80 killer cells and 200 non-killer cells. The *p*-value was determined using two-tailed Fisher's exact test.

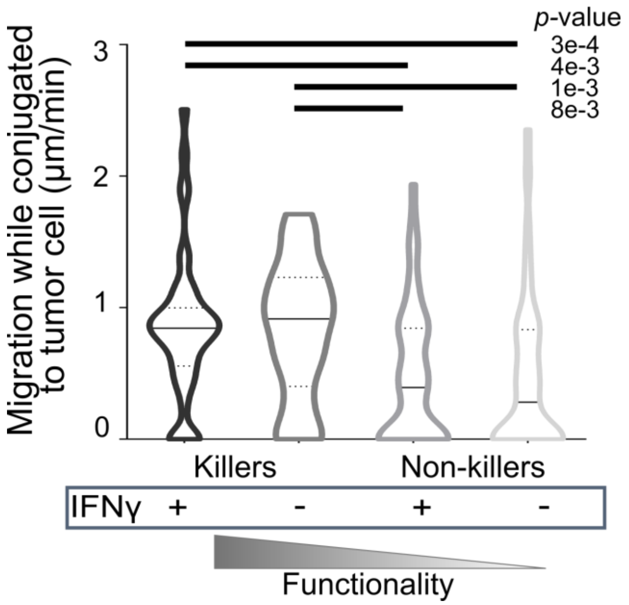

Supplementary Figure S3. T-cell migration during conjugation to tumor cells is associated with cytolytic activity in IP T cells.

Effector cells that kill targets, irrespective of whether they secrete IFN-γ, are significantly more migratory compared to non-functional effectors that do not kill or secrete IFN-γ. Data was derived from 80 killer cells and 200 non-killer cells. The bar represents the median and the dotted lines denote the quartiles. *P*-values were determined using Kruskal-Wallis non-parametric test.

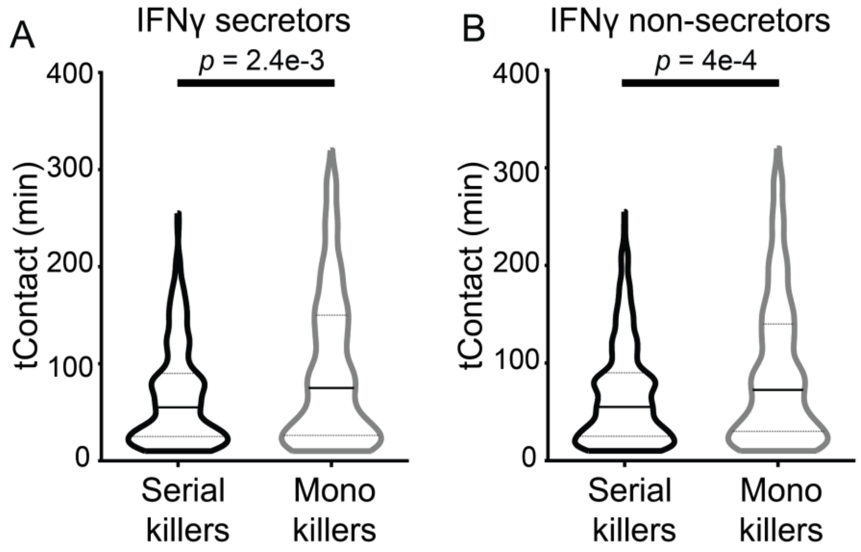

Supplementary Figure S4. Killer T cells efficiently terminate synapses with tumor cells.

(A-B) $t_{Contact}$, the cumulative duration of conjugation between T cell and tumor cell before killing, was significantly lower for serial killer 19-28z T cells in comparison to monokiller 19-28z T cells both (A) with, and (B) without IFN-γ secretion. For serial killer 19-28z T cells the $t_{Contact}$ first tumor cell killed is shown. Data was derived from 280 serial killers and 250 mono killers secreting IFN-γ, and 415 serial killers and 380 mono killers without IFN-γ secretion.

All data were derived from an E:T ratio of 1:2-5. The bar represents the median and the dotted lines denote the quartiles. For both panels, *p*-values were determined using Mann-Whitney test.

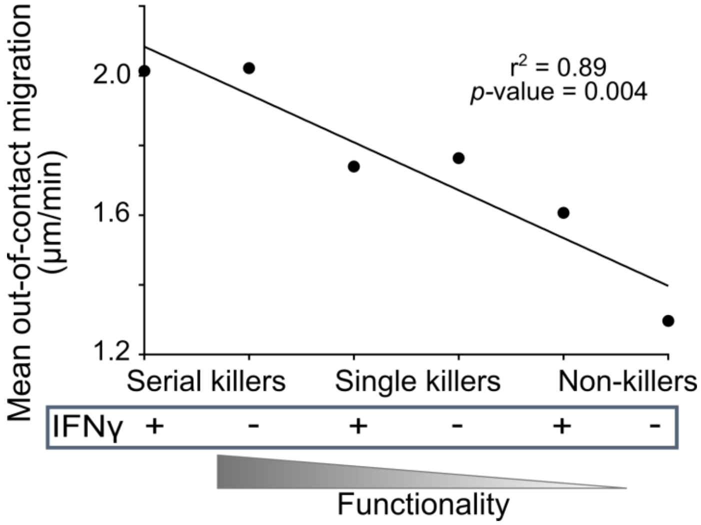

Supplementary Figure S5. Correlation between multifunctionality and mean out-of-contact T-cell migration. All data were derived from an E:T ratio of 1:2-5. *P*-value and R squared were calculated for the linear regression. From left (higher functionality) to right, each dot represents the average migration of 266, 117, 221, 113, 104 and 70 single cells respectively.

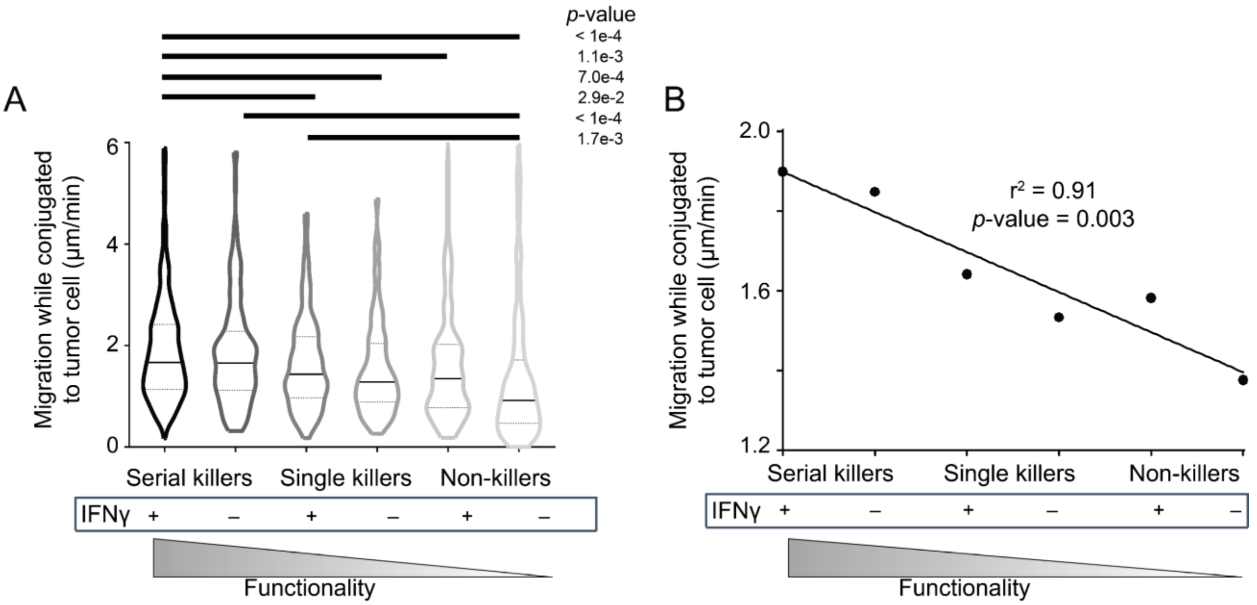

Supplementary Figure S6. Correlation between multifunctionality and mean in-contact T-cell migration during conjugation to tumor cells. At an E:T ratio of 1:2-5, average displacements of effector cells during conjugation of effector with target cells.

(A) Effector cells that kill multiple targets irrespective of whether they secrete IFN-γ, are significantly more migratory compared to non-functional effectors that do not kill or secrete IFN-γ. The bar represents the median and the dotted lines the quartiles. *P*-values were determined using Kruskal-Wallis non-parametric test.

(B) Inverse correlation between multifunctionality and mean T-cell migration during conjugation to tumor cells. *P*-value and R squared were calculated for the linear regression.

From left (higher functionality) to right, each group represents the migration of 344, 158, 276, 139, 140 and 81 single cells respectively.

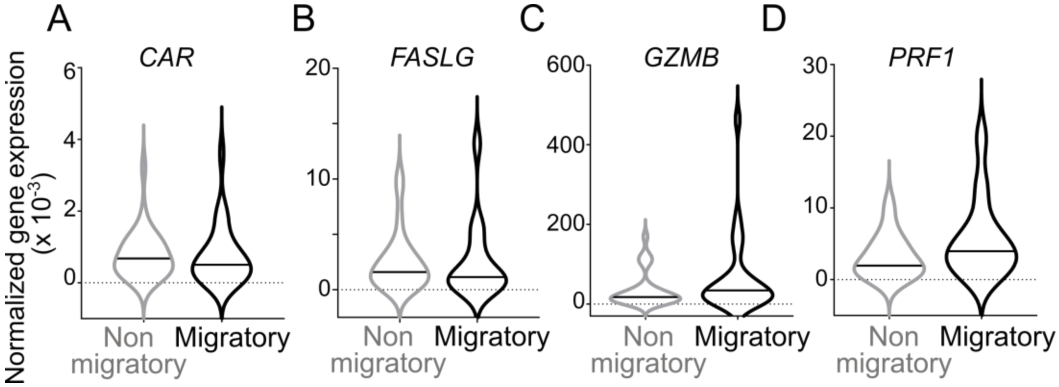

Supplementary Figure S7. Genes related to cytotoxicity are not differentially expressed between non-migratory and migratory 19-28z T cells.

(A-D) Violin plots illustrating differences between non-migratory and migratory 19-28z T cells. Data was derived from 30 migratory cells and 30 non-migratory cells. None of the genes are differentially expressed at a false-discovery rate (FDR) *q*–value < 0.1.

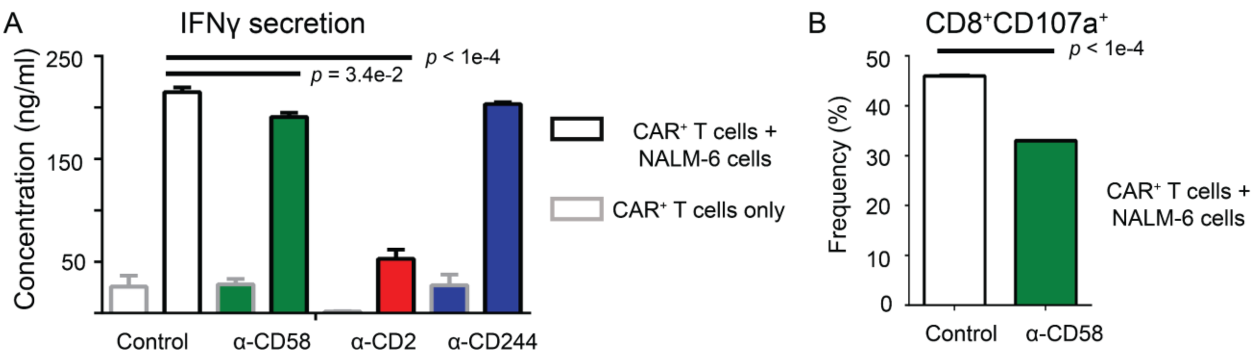

Supplementary Figure S8. Blocking the interaction between CD2-CD58 negatively impacts CAR T-cell functionality.

(A) The impact of blocking CD2, CD58, or CD244 determined by measuring cytokine secretion as determined by Intellicyt bead-based assays. These experiments were conducted on a minimum of three donor-derived 19-28z T cells. The error bars represent standard deviation, and the statistical test was one-way ANOVA.

(B) Flow cytometric assays enumerating the frequency of degranulating 19-28z T cells upon incubation with tumor cells. These experiments were conducted on a minimum of three donor-derived 19-28z T cells. Error bars represent SEM and the *p*-value was computed using a two tailed t-test.

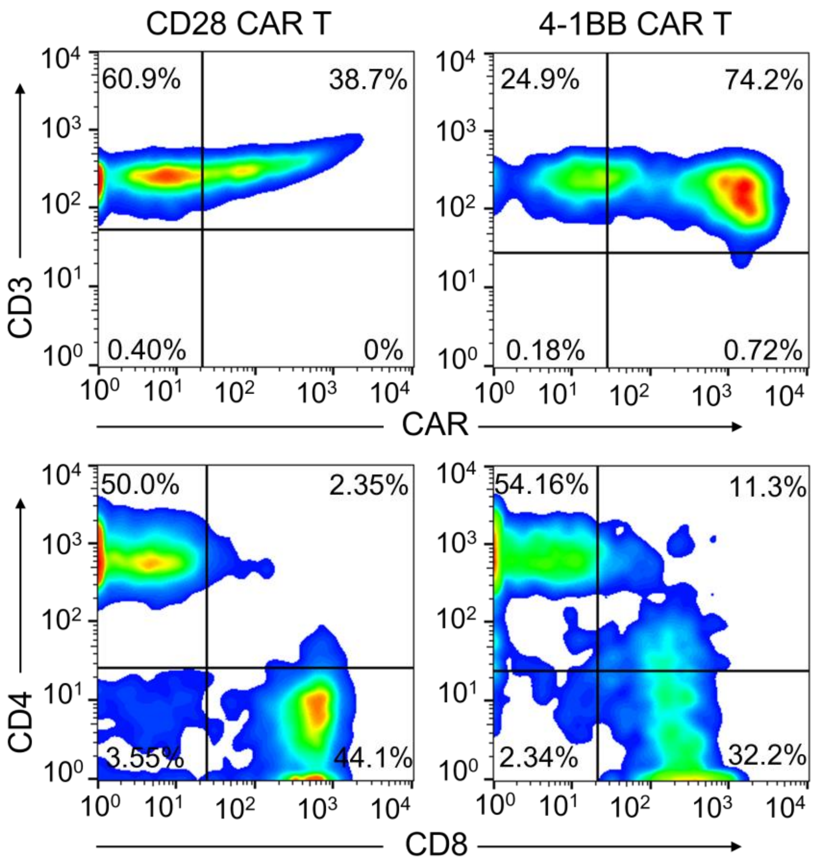

Supplementary Figure S9. Phenotype of the 19-28z and 19-41BBz CAR T cells derived from healthy donors.

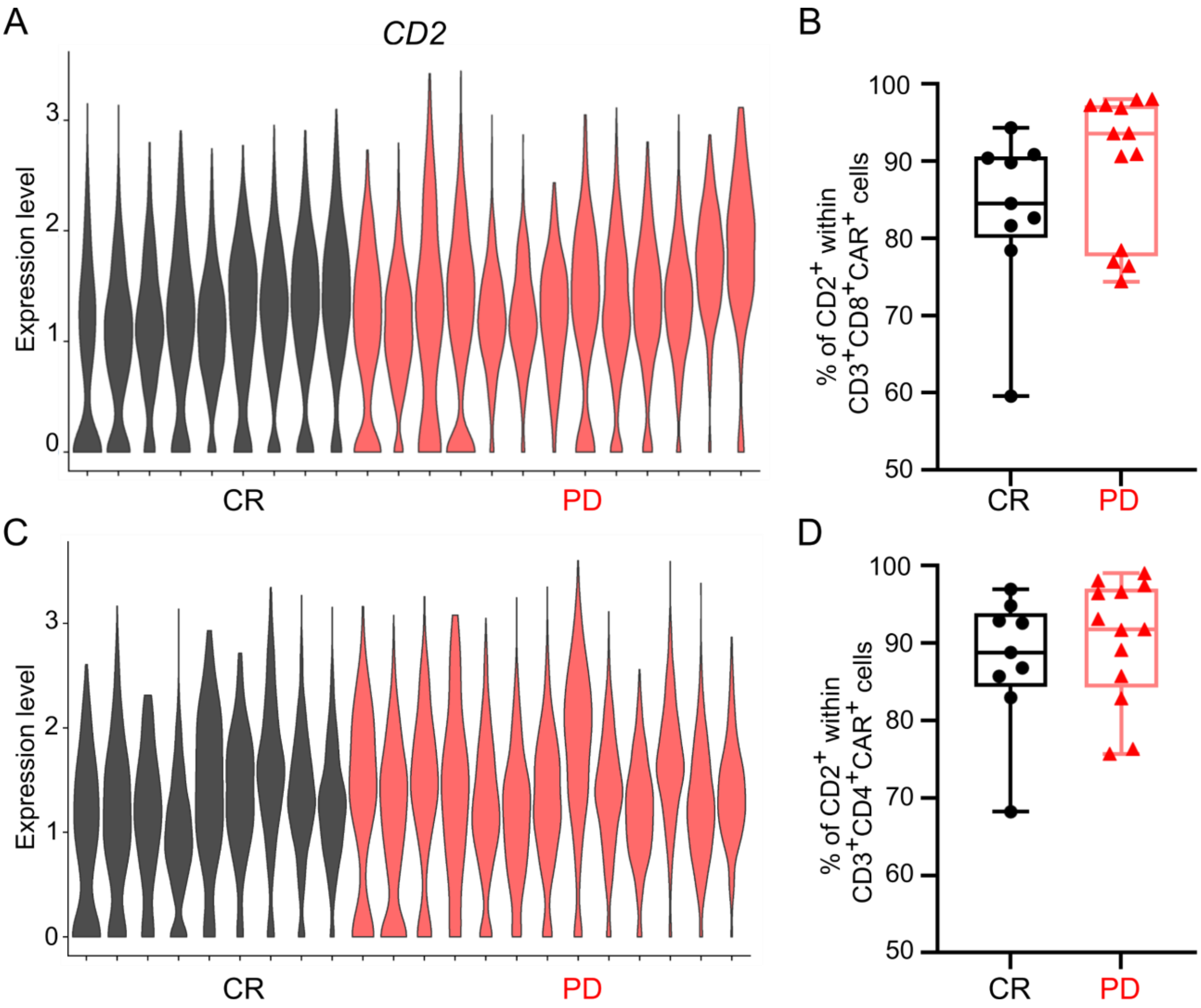

Supplementary Figure S10. ***CD2*** **expression in scRNA-seq data derived from 19-28z T cells of clinical infusion products for the treatment of LBCL.**

(A/C) Violin plots of log-transformed gene expression of *CD2* within $CD3^{+}CD8^{+}CAR^{+}$ (n=15,111) and $CD3^{+}CD4^{+}CAR^{+}$ (n=12,258) single cells in twenty-two 19-28z T cell infusion products. The scRNA-seq data is obtained from GSE151511.

(B/D) Comparison of the frequency of $CD2^{+}$ cells within $CD3^{+}CD8^{+}CAR^{+}$ and $CD3^{+}CD4^{+}CAR^{+}$ cells between complete responders (CR, n=9) and patients with progressive disease (PD, n=13). Centerline, median. Box limits, upper and lower quartiles. Whiskers, range. Mann-Whitney test *p*-value for both comparisons are not significant.

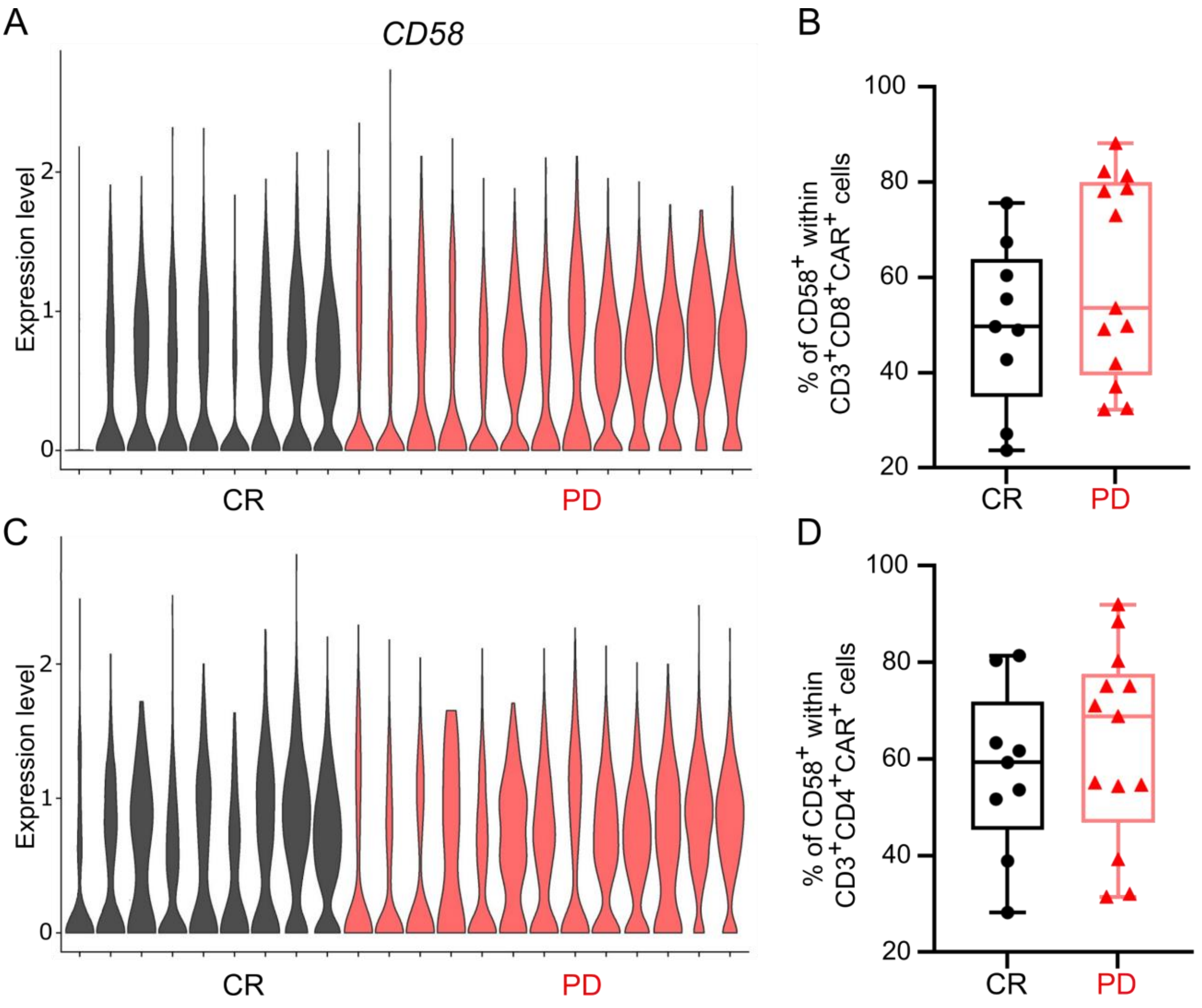

Supplementary Figure S11. *CD58* expression in scRNA-seq data derived from 19-28z T cells of clinical infusion products for the treatment of LBCL.

(A/C) Violin plots of log-transformed gene expression of CD58 within CD3$^+$CD8$^+$CAR$^+$ (n=15,111) and CD3$^+$CD4$^+$CAR$^+$ (n=12,258) single cells in twenty-two 19-28z T cell infusion products. The scRNA-seq data is obtained from GSE151511.

(B/D) Comparison of the frequency of CD58$^+$ cells within CD3$^+$CD8$^+$CAR$^+$ and CD3$^+$CD4$^+$CAR$^+$ cells between complete responders (CR, n=9) and patients with progressive disease (PD, n=13). Centerline, median. Box limits, upper and lower quartiles. Whiskers, range. Mann-Whitney test *p*-value for both comparisons are not significant.

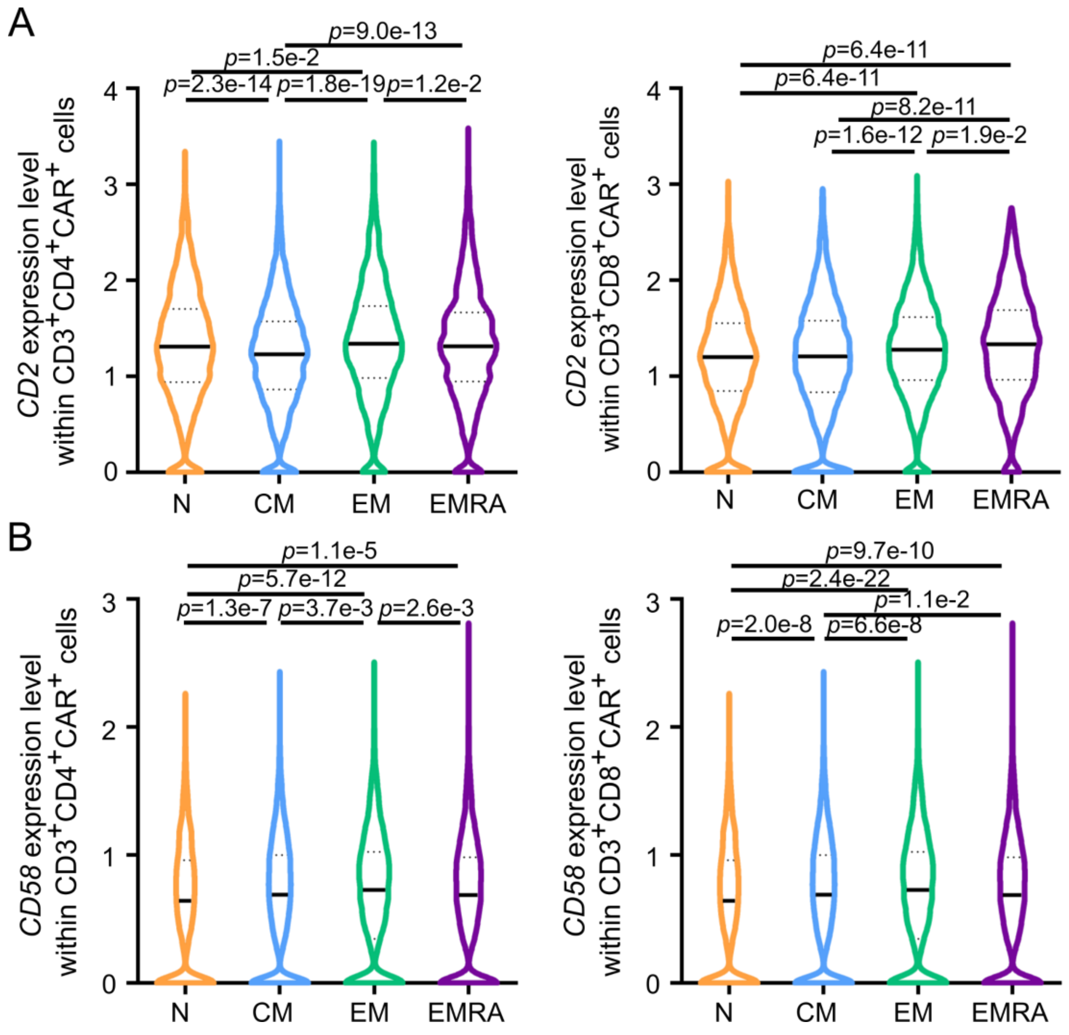

Supplementary Figure S12. *CD2* and *CD58* expression within T cell subsets in scRNA-seq data derived from 19-28z T cells of clinical infusion products for the treatment of LBCL.

(A) Violin plots of log-transformed gene expression of *CD2* within $CD3^+CD4^+CAR^+$ and $CD3^+CD8^+CAR^+$ single cells in four T cell subsets. N: naïve (4111 cells), CM: central memory (5517 cells), EM: effector memory (2352 cells), EMRA: effector memory re-expressing CD45RA (3501 cells). The scRNA-seq data is obtained from GSE151511.

(B) Violin plots of log-transformed gene expression of *CD58* within $CD3^+CD4^+CAR^+$ and $CD3^+CD8^+CAR^+$ single cells in four T cell subsets. All *p*-values were computed using Wilcoxon test.
